# The Number of Surgeons Using Superior Capsular Reconstruction for Rotator Cuff Repair Is Declining

**DOI:** 10.1016/j.asmr.2022.10.002

**Published:** 2022-11-17

**Authors:** Abby C. Hankins, Justin W. Griffin, John P. Taliaferro, Brian C. Werner, Kevin F. Bonner

**Affiliations:** a Eastern Virginia Medical School, Norfolk, Virginia, U.S.A.; b Jordan-Young Institute, Virginia Beach, Virginia, U.S.A.; c Orthopaedic Research of Virginia, Richmond, Virginia, U.S.A.; d Department of Orthopedic Surgery, University of Virginia, Charlottesville, Virginia, U.S.A.

## Abstract

**Purpose:**

To investigate surgeon preferences for graft use, including biologic augmentation and superior capsular reconstruction (SCR) associated with surgical treatment of rotator cuff repair (RCR).

**Methods:**

A 26-question survey was completed by arthroscopic shoulder surgeons. Surgeon demographics were evaluated. Surgeons were queried about shoulder arthroscopic graft use and rationale then responses were analyzed based on demographics.

**Results:**

In total, 260 surgeons completed the survey. Fifty-one percent of surgeons reported a decrease in the volume of SCR use in the past 5 years. Less than 3% of surgeons used SCR in >90% of irreparable RCR cases, compared with 38% using SCR in <10% of irreparable cases (*P* < .05). Surgeons performing >100 RCR annually (42%; *P* < .05) and those employed in the hospital setting (44%; *P* < .05) reported an overall increase in the use of SCR. More international surgeons (67%) decreased their use of SCR compared with U.S. surgeons (44%; *P* < .05). In contrast, bioinductive graft use is generally on the rise, with 48% of surgeons reporting increased use since first use, although used in <10% of cases by 54% of surgeons. Sixty-eight percent of surgeons performing >100 RCRs annually used bioinductive grafts (*P* < .05). Fewer international surgeons (30%) performed biologic augmentation (*P* < .05). Suboptimal published outcomes (40%) and no perceived patient benefit (40%) were most cited for decreased SCR use. Surgeons reporting increased use cited improved personal patient outcomes (72%).

**Conclusions:**

Arthroscopic surgeons report decrease in volume of SCR use in the past 5 years. Surgeon’s personal experience of patient outcome and suboptimal published results were the strongest factors impacting decision-making. In contrast, bioinductive graft use is increasing. However, most surgeons use these grafts in a relatively small percentage of cases.

**Clinical Relevance:**

Evaluation of scientific data, personal experience, and influences on surgical practice will give a comprehensive understanding of current RCR practices.

Rotator cuff tears are the most common shoulder pathology for which patients seek care and undergo surgery.^1^ Unfortunately, many massive tears are only partially repairable or irreparable at the time of surgery. In addition, healing failure and recurrent tears are common, especially in the setting of larger tears or poor-quality tissue.^2^ Decision-making and optimal treatment for these irreparable tears and high-risk repairs continue to be debated. Several emerging techniques, including superior capsular reconstruction (SCR) and biologic graft augmentation, have been introduced as treatment options over the past several years.^3^, ^4^, ^5^

SCR using a fascia lata (FL) autograft to attach medially to the superior glenoid and laterally to the greater tuberosity in order to stabilize the glenohumeral joint and restore function has been described.^6^^,^^7^ Although initial reports with FL autograft out of Japan were excellent,^7^^,^^8^ more recent reports by other surgeons show variable success.^9^, ^10^, ^11^, ^12^ In the United States, human dermal allografts are the grafts used predominantly for SCR,^13^ compared with the thicker autograft.^7^^,^^14^^,^^15^

Similarly, rotator cuff repair (RCR) using biologic and/or structural augmentation (onlay) and interpositional grafts (IG), also referred to as “bridging grafts,” have been introduced as scaffolding methods to address the challenges of suboptimal healing, poor tissue quality, and irreparable defects. Extracellular matrices, specifically acellular human dermal allografts, as well as engineered collagen or synthetic grafts, are becoming common biologic scaffolds employed to aid in bolstering clinical outcomes,^16^ proposed to potentially reduce rates of retears and improve healing. IG are implants used to span an irreparable defect or gap between the residual irreparable tendon and bone footprint,^17^ whereas biologic augmentation is an implant placed on the bursal side of the RCR construct or alternatively at the interface between the tendon and bone (enthesis site). The general rationale is to attempt to enhance healing, give rise to additional collagen, and provide a more robust resultant tendon, although most currently do not provide mechanical reinforcement.^18^ The key difference of IG compared with SCR is that IG attempts to provide a structural graft and fill in a defect between the dynamic residual tendon and the bone interface, whereas SCR is fixed from the glenoid to the tuberosity and aims to restore joint kinematics and stability.^15^^,^^19^ Clinical results in RCR have been variable with these biologic products, although there have been some early favorable reports with lower levels of evidence.^20^, ^21^, ^22^ There is basic science and early clinical reports to support SCR and biologic augmentation.^6^^,^^9^^,^^20^, ^21^, ^22^ However, they are being heavily marketed with a lack of clear indications and conclusive outcome improvement to perhaps justify the associated increased costs and technical challenges.^23^

SCR and biologic graft augmentation use among arthroscopic shoulder surgeons is poorly understood. It is uncertain whether decision-making regarding graft usage is influenced primarily by surgeon training and background or other factors. Evaluation of scientific data, personal experience, colleague, and industry influence may contribute to a surgeon’s decision-making regarding if and when to introduce and indicate the use of biologic grafts and SCR. The purpose of this study is to define current use in RCR regarding SCR, biologic graft augmentation and graft preference of surgeons, as well as explore factors associated with a potentially changing landscape. We also hypothesized that that the rate at which surgeons use SCR would be on the decline. We anticipated graft choice and use would be impacted by surgeon demographics, such as years in practice, volume of rotator cuffs performed annually, region of practice, practice setting, and sports medicine certification.

## Methods

A cross-sectional study of members of the Arthroscopic Association of North America (AANA) was performed. The study was to include board-certified or board-eligible orthopaedic surgeons from the United States, as well as internationally. The survey was dispersed to AANA surgeons who either indicated shoulder as their primary or secondary joint or attended one of the AANA shoulder courses between the time frame of 2001 to 2017. This study was approved by the Eastern Virginia Medical School Institutional Review Board (approval date: April 23, 2021, no. 21-04-XX-0105).

A 26-question survey was distributed to approximately 3,700 orthopaedic surgeons via an e-mail link to Survey Monkey. The survey was distributed by AANA marketing team via listserv. All responses were anonymous, and the authors did not have access to surgeon names or e-mail addresses. The survey was distributed in May 2021, with one reminder e-mail sent halfway through the study period. Survey submission required answering all demographics, but questions regarding graft preferences were optional. Table 1 contains a list of survey questions.

**Table 1:** Comprehensive View of Questions That Comprised the Survey Sent to AANA Surgeons

Survey Questions
1. Are you board-certified?
2. Are you board-eligible?
3. Are you certified in Sports Medicine?
4. Have you completed a Sports Medicine or Shoulder & Elbow Fellowship?
5. In what region do you currently practice?
6. How many years have you been practicing as an Orthopaedic Surgeon?
7. What is your primary practice setting?
8. Approximately how many rotator cuff repairs do you perform in 1 year?
9. What percentage of your surgical practice would you consider related to rotator cuff surgery?
10. Rank the following factors on how likely they are to influence your decision-making when treating rotator cuff tears (with 1 being most likely to influence and 6 being least likely): training/mentorship, CME courses, industry courses and marketing, input from colleagues, institutional policy, personal experience
11. How would you define your routinely performance of rotator cuff repair? >95% of cases arthroscopic, >95% of cases open, smaller tears arthroscopic and larger, and more challenging tears open
12. Have you performed SCR in your practice in the past 5 years?
13. In what percentage of your “irreparable” rotator cuff procedures do you currently perform SCR?
14. What is your current graft preference for SCR? autologous fascia lata, dermal allograft, autologous biceps tendon, other (please specify)
15. Compared with 2-5 years ago, how would you characterize your use of SCR?
16. Reasons for decreased SCR use: complexity of procedure does not justify outcomes, no perceived benefit to personal patient outcomes to justify use, complications related to the procedure, cost, published or presented reports of suboptimal results or nonhealing, unpublished reports of suboptimal results, need for data on long-term outcomes, institution or ambulatory service center does not allow, other (please specify)
17. Reasons for same or increased SCR use: improved reported patient outcomes (literature or CME courses), improved personal patient outcomes (your own experience), influence of industry courses or marketing improved results, colleagues communicating promising clinical results, other (please specify)
18. Relative to 3-5 years ago, your general inclination is that surgeons around you are using SCR: never, almost never, much less, somewhat less than 3-5 years ago, same as 3-5 years ago, somewhat more now than 3-5 years ago, much more than 3-5 years ago
19. Have you or do you currently use biological rotator cuff repair augmentation using a graft for the purpose of improving healing or bridging a rotator cuff defect that cannot be repaired?
20. If you use a biologic augmentation graft for tissue repair, what percentage of your rotator cuff procedures do you use a biological graft for its potential bioinductive properties?
21. If you use a biological graft in your practice as a bridging graft, what percentage of your rotator cuff procedures that you cannot fully repair but have a residual defect do you use a biologic “bridging” graft?
22. When using biologic rotator cuff augmentation of a rotator cuff repair, where do you prefer to place the graft?
23. Compared with when you first started using a biological graft augmentation to enhance healing (bioinductive) for your rotator cuff repairs, how would you characterize your current use and use in the near future?
24. Why do you not use biologic rotator cuff augmentation for these purposes?
25. Assuming there is not substantial arthritis, if you have an irreparable or partially repairable tear, what is your typical surgical treatment of choice for a 55-year-old patient’s first shoulder surgery in addition to a biceps tenodesis/tenotomy? debridement with or without partial repair, SCR, interpositional graft (i.e., graft that bridges from the residual rotator cuff to the tuberosity), reverse shoulder arthroplasty
26. Assuming there is not substantial arthritis, if you perform a revision rotator cuff repair after a previously failed prior rotator cuff repair, what is your typical surgical treatment of choice for a 55-year-old patient’s first shoulder surgery in addition to a biceps tenodesis/tenotomy? debridement with or without partial repair, SCR, interpositional graft (i.e., graft that bridges from the residual rotator cuff to the tuberosity), reverse shoulder arthroplasty

AANA, Arthroscopic Association of North America; CME, Continuing Medical Education; SCR, superior capsular reconstruction.

The primary outcome was to understand current practices for RCR, biologic graft augmentation, and graft preference of surgeons. Secondary aims explored factors associated with their use and preference. Respondents were first asked to rank the following factors in how they influence their surgical decision-making: training/mentorship, Continuing Medical Education courses, industry courses and marketing, input from colleagues, institutional policy, and personal experience. Surgeons were then asked about their preferred method for performing RCR (arthroscopic vs open). The next 7 questions answered by shoulder surgeons assessed their usage of SCR, both personal and their inclination of other surgeons. Answers to surgeon personal preference for SCR were then further evaluated to determine reason for increased or decreased use. The following 6 questions addressed surgeon preference for bioinductive grafts and their methods of use.

Surgeon and practice demographics were collected and associated with graft use and preference. Surgeon demographics included board certification, completion of a sports medicine certification, and completion of a sport or shoulder fellowship. Practice characteristics included practice region, years in practice, practice setting, and volume of RCRs performed per year. The final aspect of the survey presented 2 scenarios of a patient of the same age without substantial arthritis depicting differing rotator cuff injuries, and surgeons were queried on their choice of surgical treatment for each case.

Deidentified data were stored by Survey Monkey and compiled by the AANA marketing team. Microsoft Excel (Microsoft, Redmond, WA) was used to organize and plot data. Analysis of variance comparisons were performed to compare results between characteristic groups, assuming normal population distribution, independence of data and equal variances. For all statistical comparisons, a *P* value ≤.05 was considered statistically significant.

## Results

Two hundred sixty total arthroscopic surgeons responded to the survey. Respondent personal and practice demographics are displayed in Table 2.

**Table 2:** Surgeon Respondents’ Personal and Practice Demographics

Demographic	N = 260
Board-certified	209 (80.4%)
Sports Medicine CAQ	157 (60.3%)
Sports or Shoulder Fellowship	206 (79.2%)
Practice region	
West	32 (12.3%)
Southwest	24 (9.2%)
Midwest	43 (16.5%)
Northeast	41 (15.8%)
Southeast	34 (13.1%)
International	68 (26.2%)
Years in practice	
<5	30 (11.5%)
5 to <10	40 (15.4%)
10 to <20	71 (27.3%)
20 to 30	71 (27.3%)
>30	35 (13.5%)
Practice setting	
Academic	33 (12.7%)
Hospital employed	64 (24.6%)
Private practice	141 (54.2%*)*
Military	5 (1.9%)
Rotator cuff repairs per year	
<50	97 (37.3%)
51-100	72 (27.7%)
>100	78 (30.0%)

CAQ, Certificate of Added Qualifications.

### Rotator Cuff Preferences

Surgeons were queried regarding factors influencing their clinical decision-making when addressing rotator cuff tears. It was found that training and mentorship (46%), followed by personal experiences (36%) were most likely to influence decision-making, whereas institutional policy was the least likely factor to influence (71%). Regarding preferred technique, nearly 91% of surgeons reported using arthroscopic repair in greater than 95% of their cases, with 4% of surgeons performing open repair in greater than 95% of cases. The remainder of surgeons reporting using arthroscopic technique in smaller tears, while using open on larger, more complex and challenging tears.

### Superior Capsular Reconstruction

**Fig 1:**
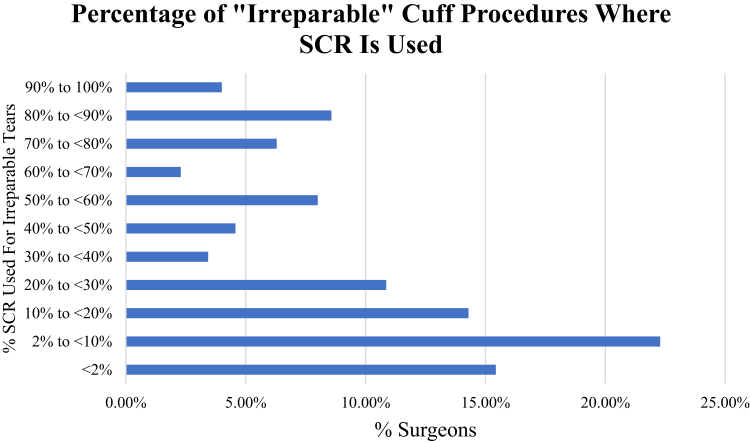
Surgeons were queried on what percentage of their cases where the rotator cuff tear was considered "irreparable" that they performed SCR. A total of 4% of surgeons who reported using the SCR technique used SCR in greater than 90% of their irreparable RCR cases, as compared with 38% reporting they used SCR in less than 10% of their irreparable cases. (RCR, rotator cuff repair; SCR, superior capsular reconstruction.)

Of all respondents, 71% of surgeons reported using SCR at least once in their practice setting within the past 5 years. Surgeons were queried about their use of SCR in irreparable procedures (Fig 1), and it was found that 4% of surgeons who reported using SCR technique used SCR in greater than 90% of their irreparable RCR cases, as compared with 38% reporting they used SCR in less than 10% of their irreparable cases. For surgeons using SCR, dermal allografts led in graft preference choice (76%), followed by autologous biceps tendon (12%) and autologous FL (11%). One percent of surgeons reported having other graft preference from the aforementioned options.

**Fig 2:**
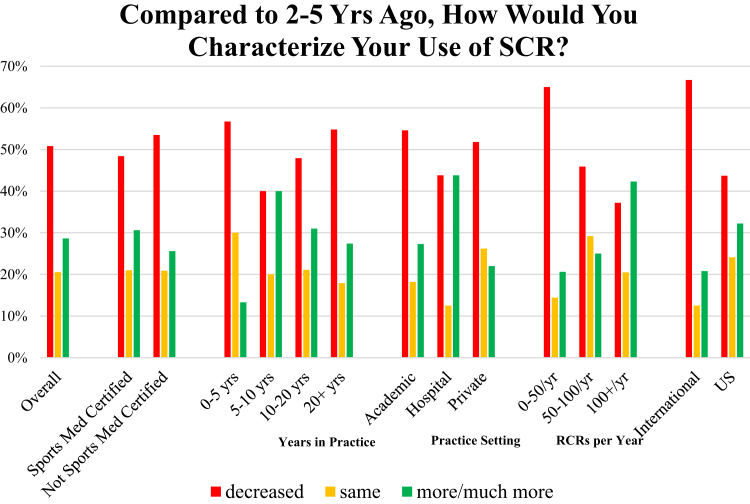
The survey aimed to evaluate a 5-year period of SCR use. It was found that, overall, the majority of surgeons (38%) responded they never or almost never use SCR. The exception was surgeons performing greater than 100 RCR/year and those employed in the hospital setting reportedly increased use of SCR at 42% and 44%, respectively. (RCR, rotator cuff repair; SCR, superior capsular reconstruction.)

After establishing arthroscopic shoulder surgeon preferences for SCR and grafting, the subsequent aim of the study was to determine whether SCR use preferences changed over the past 2 to 5 years (Fig 2) and, if so, what were factors driving this change. Overall, decreased SCR use was reported in the past 2 to 5 years, with 51% of surgeons reporting decreased SCR use, 20% reporting the same use, and 29% reporting increased use. Surgeons in high-volume settings, performing greater than 100 rotator cuff repairs per year, reported increased SCR use compared with surgeons performing fewer than 50 RCRs per year (*P* < .05). This suggests that although the volume of surgeons performing SCR is generally on the decline, surgeons performing a greater volume of RCR are increasing volume of SCR and therefore the total number of SCRs performed may actually be increased over the past 5 years. Surgeons were then asked to establish rationale for their preferences, justifying reasoning for same or increased use of SCR or decreased use of SCR. For surgeons reporting decreased SCR use, leading rationales included published or presented reports of suboptimal results or nonhealing (40%) and no perceived benefit to personal patient outcomes to justify use (40%). Orthopaedists reporting increased use of SCR cited their own personal patient outcomes as the leading rationale (72%).

### Bioinductive Grafts

**Fig 3:**
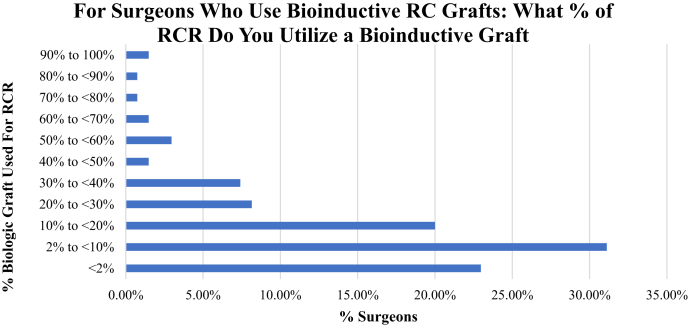
Surgeons who reported use of biologics grafts were queried on what percentage of their RCRs they used biologic matrices. A total of 1.48% of surgeons used biologics in greater than 90% of their cases, while 54% used in fewer than 10% of cases. (RC, rotator cuff; RCR, rotator cuff repair.)

**Fig 4:**
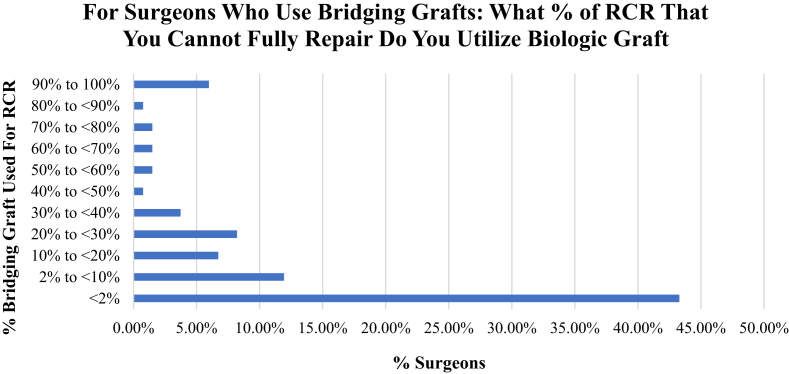
Surgeons reporting use of bridging grafts were asked what perfect of RCR cases that are not fully repairable would they use biologic grafting. In total, 6% of surgeons would use in greater than 90% of cases, whereas 55% would use in fewer than 10% of cases. (RCR, rotator cuff repair.)

Of all respondents, 56% reported having currently or in the past used biological RCR augmentation using a graft. Surgeons performing fewer than 50 RCRs per year (41% of surgeons) were less likely to use bioinductive grafting compared with their counterparts performing greater than 100 RCR per year (68% of surgeons; *P* < .05). When asked to characterize their current and future use of bioinductive grafts compared with when they first started using the technique, 48% of surgeons overall reported increased use of bioinductive grafts. For surgeons who are using bioinductive grafts, they were queried on what percentage of their rotator cuff procedures they used a biological graft for its potential bioinductive properties (Fig 3). Fewer than 3% of surgeons used bioinductive grafts in greater than 70% of their RCR procedures. Surgeons who reported using bioinductive grafts as bridging grafts were followed up by being asked what percentage of their rotator cuff procedures that they cannot fully repair but have a residual defect do they use a biologic “bridging” graft (Fig 4). Six percent of surgeons used bridging grafts in greater than 90% of their RCR procedures with residual defects. Fifty-five percent of surgeons reported using bridging grafts in fewer than 10% of same procedures. Surgeons who did not use bioinductive grafting were asked to explain their rationale. It was found that cost of the procedure was the leading reason for 51% of surgeons, followed by need for data on long-term outcomes (36%), and no perceived benefit to patient outcomes in their personal experiences (36%).

**Fig 5:**
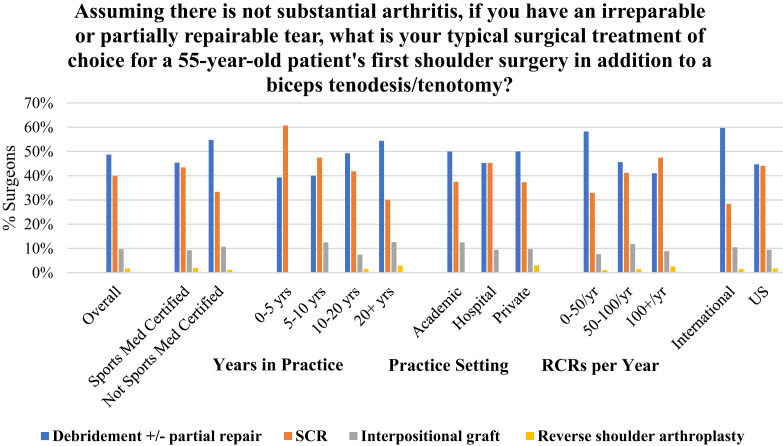
Surgeons were presented with a case scenario of an irreparable or partially repairable tear in a 55-year-old patient’s first shoulder surgery without substantial arthritis and asked their surgical method of choice. Overall, 49% of surgeons chose debridement with or without partial repair and 40% chose SCR. Those performing greater than 100 RCR/year (47%) and those in practice less than 5 years (61%) were more likely to use SCR. (RCR, rotator cuff repair; SCR, superior capsular reconstruction.)

**Fig 6:**
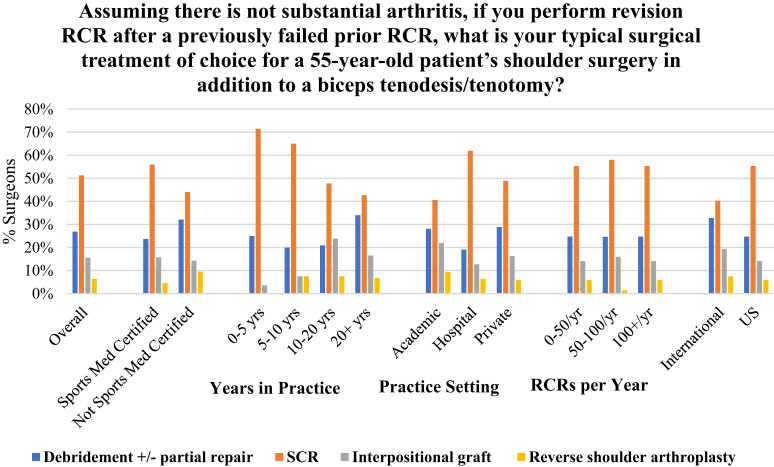
Surgeons were presented with a case scenario in which they were asked what revisional RCR they would perform after a previously failed RCR in a 55-year-old patient without substantial arthritis. Overall, 51% of surgeons chose SCR, followed by 27% choosing debridement with or without partial repair. SCR was most selected procedure among all demographics, although surgeons in practice fewer than 5 years (71%) and those employed by hospital (62%) were most likely to perform SCR. (RCR, rotator cuff repair; SCR, superior capsular reconstruction.)

Two proposed scenarios at the conclusion of the survey evaluated theoretical clinical treatment preferences for 2 RCR cases. The first asked for preferred treatment choice for an irreparable or partially repairable tear in a 55-year-old patient without substantial arthritis who has not had previous surgery (Fig 5). Debridement, with or without partial repair, was the leading choice (49%), followed by SCR (40%). It was found that surgeons in practice for fewer years preferred SCR over debridement, with 60% of surgeons having 0 to 5 years of experience choosing SCR compared with 48% of surgeons with 5 to 10 years preferring SCR. Surgeons with a greater volume of RCR repairs (>100 RCR/year) were more likely to choose using SCR in this scenario (47%) compared with lower-volume surgeons, with 41% of surgeons performing 51 to 100 RCR per year choosing SCR and 33% of surgeons performing fewer than 50 RCR per year choosing SCR. The second scenario asked for treatment preference for a RCR revision surgery after a previously failed RCR in a 55-year-old patient without substantial arthritis (Fig 6). Overall, SCR was the leading choice among all demographics, with 51% of surgeons preferring SCR, followed by debridement at 27%. Similar to the previous question, it was found that a greater number of surgeons in practice for fewer years preferred SCR over debridement compared with their counterparts with more years in practice.

## Discussion

In this study, we found that that an increased percentage of arthroscopic surgeons decreased their volume of SCRs performed over the past 5 years. The factors driving these changes include endorsing a lack of perceived benefit to the patient to justify use and reports of suboptimal results. Although initial studies showed promising results, with SCR improving pain and overall function for patients while preventing progression to arthropathy,^7^^,^^9^^,^^14^^,^^24^ more recent reports document graft healing issues, technical difficulty, reoperation, and need for subsequent revision or conversion to arthroplasty.^25^

Studies report excellent clinical results after SCR using primarily a thick-folded autologous FL graft.^7^^,^^8^^,^^26^ In an effort to minimize donor-site morbidity^12^^,^^16^ and expedite surgery, the use of acellular dermal allografts has become a popular alternative^15^ to autografts in the United States.^14^^,^^27^ A recent systematic review of 14 studies commonly reported complications of SCR using dermal allografts, such as graft retear due to rupture of graft, anchor displacement, revision, reoperation, and infection.^12^ Surgeons in our study reported their rationale for not using or decreasing the use of SCR to primarily be due to published reports of suboptimal outcomes and no perceived benefit to their personal patient outcomes. In addition, surgeons reported decreasing SCR use due to other factors, such as lack of long-term outcome data, complexity of procedure, and complications related to operation. As data continue to be born out in the literature, with some reporting suboptimal outcomes or disappointing healing rates with the dermal allograft, the complexity and cost of the procedure may be leading surgeons to pursue alternative treatment options for an irreparable tear, such as partial repair, debridement, addressing the biceps tendon and concomitant pathology, tendon transfers, subacromial balloon interposition, and reverse shoulder arthroplasty.^28^^,^^29^

Biologic adjuvants are a current focus in an effort to enhance rotator cuff healing and clinical results, especially in the setting of poor-quality tissue or larger tears that have historically had high failure rates of healing.^18^^,^^23^ Our study shows that the majority of surgeons surveyed (56%) have used biologic augmentation to some degree in their practice. Of these surgeons, nearly one half (48%) have increased their use of these products since first use. Based on our data, relatively few surgeons regularly use grafts for either their bioinductive properties or as a “bridging” graft. Ultimately, surgeons reported the cost of these products to be the most common limiting factor, followed by the lack of long-term outcome data. Some surgeons report concerns due to suboptimal published outcomes. More studies are needed to assess the reliability and success of biologic graft augmentation in RCR.^30^ Since introduced, some of these biologic grafts have been shown to adversely affect outcomes and have served to elicit foreign body reactions,^31^ whereas some more recent studies show potential benefit, especially with greater-risk repairs.^32^ A 2022 study performed on existing literature suggests that the increased cost of graft augmentation proves to be a cost-effective procedure due to increased quality-adjusted life years across 10 years when compared with RCR without graft augmentation.^33^ We expect that if data continue to prove to be beneficial to patients in the long term, then surgeons may justify the cost and complexity of procedure.

This study aimed to evaluate not only SCR and graft use but shoulder surgeons’ latest approaches to primary and revision operations for 2 irreparable rotator cuff tear scenarios. The first case presented a relatively young patient without substantial arthritis presenting with an irreparable or partially reparable rotator cuff tear without a previous history of cuff repair, whereas the second scenario presented a patient of the same age without substantial arthritis presenting for revision after failed RCR. Regarding the patient with an irreparable or partially reparable tear without previous RCR, it was found that surgeons overall were most likely to perform debridement with partial repair as a primary procedure for an irreparable tear without arthritis (Fig 5), followed by SCR. However, surgeons performing the greatest volume of RCRs annually, greater than 100 RCRs per year, and those with fewer than 10 years of practice, preferred SCR in this case. In the patient presenting after failed RCR, in addition to a biceps tenodesis/tenotomy, surgeons preferred SCR as the treatment of choice (Fig 6). These results were consistent across years in practice, practice setting, RCR volume, certification, and geographic location.

**Fig 7:**
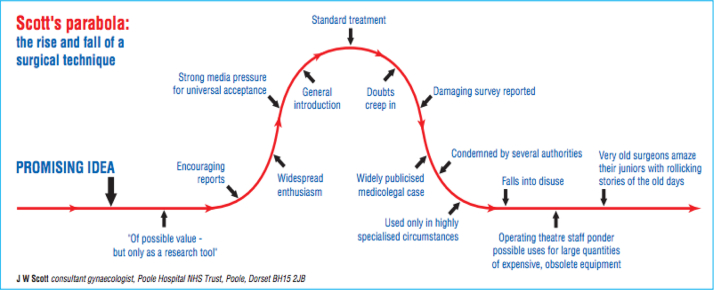
“Scott’s Parabola: The Rise and Fall of a Surgical Technique”^34^ explains the shift of how a procedure goes from encouraging data and a promising idea that drives it to be the standard of care to a procedure that is rarely used after reports of complications and suboptimal data. Scott’s parabola can possibly account for the changing prospects of SCR revealed in this survey. (SCR, superior capsular reconstruction.)

“Scott’s parabola: the rise and fall of a surgical technique” (Fig 7^34^) may provide additional insight into the results revealed in this study.^35^ Scott’s parabola graphically represents the increased use of a technique or procedure when there is initial great promise and encouraging results, leading it to be perceived as the standard of care, followed by the decrease in use after negative reported outcomes come to light. SCR was a promising idea composed in 1993 and first performed in the United States as a dermal allograft in 2014. If true, then this phenomenon may help explain why the perceived use of SCR was reportedly greater than the actual use of SCR in this study, as is it possible publications have not yet reflected the decreased use of SCR among surgeons.

In the years since introduction, SCR has gained popularity remarkably due to reports of encouraging patient outcomes.^9^^,^^10^^,^^28^ As time has gone on, published data continue to come out reporting both the positive patient-reported outcomes of SCR but also the challenges of SCR, including retears and nonhealing.^12^ In this study, surgeons refer to published and personal outcomes, as well as the complexity of the procedure, as leading reasons to decreased use. As with many procedures, the more technically challenging, time-consuming, and expensive, the greater benefit that needs to achieved in order to be justified and used. As reported in the literature and by arthroscopic respondents, the cost of SCR presents a socioeconomic issue that often effects decision-making.^36^ In addition, some reports emphasize that less complex and costly procedures are reasonable options to initially address a massive RC tear, especially as a primary procedure, such as partial repair that has some literature reporting only 5% of patients necessitating revision or reoperation.^37^ That being said, many patients with massive irreparable tears do not always do well with partial repair, hence why this is still a clinical dilemma for many patients and surgeons.

Overall, there is no perfect solution or procedure that has shown clinical superiority for patients with irreparable rotator cuff tears, but rather options that have shown success in certain populations of patients. It is ultimately a combination of surgeon preference and ever-changing literature that drives decision-making and surgical choice.

### Limitations

Limitations to this study include an overall low response rate at 260 responses, which was 7% of the total pool (3,700) of surgeons to whom the survey was e-mailed, potentially leading to bias that overemphasizes the results reported in this study. We are not sure how many surgeons received or opened the e-mail invitation. This may not be a fair representation of members or surgeons regarding their perspectives regarding these complex topics. Fewer numbers of respondents in subgroups (such as surgeons in the Southwest, those in practice fewer than 5 years, and military surgeons) may provide limitations in comparisons. Other recently published survey studies regarding orthopaedic surgeons’ preferences report low response rate as a potential confounder as well.^38^^,^^39^ In addition, only orthopaedic surgeons who are AANA members were queried, potentially resulting in response bias, as this one organization may not be entirely representative of all orthopaedic shoulder surgeons. In terms of reasons for graft preference and use, there are likely other factors that influence surgeon decision-making for which we were not able to account in this study. We did not ask for potential confounding factors that may affect decision-making and opinions, such as conflict of interest related to RCR, SCR, or biologic graft use.

## Conclusions

Arthroscopic surgeons report a decrease in the volume of SCR use in the past 5 years. Surgeon's personal experience of patient outcome and suboptimal published results were the strongest factors impacting decision-making. In contrast, bioinductive graft use is increasing. However, most surgeons use these grafts in a relatively small percentage of cases.

## Supplementary Data

ICMJE author disclosure forms
