# Supplementary material for: The Number of Surgeons Using Superior Capsular Reconstruction for Rotator Cuff Repair Is Declining
Source: Arthrosc Sports Med Rehabil. 2022 Nov 17;4(6):e2089–98. doi: 10.1016/j.asmr.2022.10.002 (PMC9791834; doi:10.1016/j.asmr.2022.10.002)
Supplement: ICMJE author disclosure forms [file mmc1.pdf]

Arthroscopy: The Journal of Arthroscopic and Related Surgery

— Instructions —

ICMJE Form for Disclosure of Potential Conflicts of Interest

Each author of the manuscript must separately complete and save this form using his or her name in the file name. Each author's completed form must then be uploaded with the manuscript.

The purpose of this form is to provide readers of your manuscript with information about your other interests that could influence how they receive and understand your work. The form is in four parts:

Section 1. Identifying information

Enter your full name and provide the manuscript title.

Section 2. The work under consideration for publication

This section asks for information about the work that you have submitted for publication. The time frame for this reporting is that of the work itself, from the initial conception and planning to the present. The requested information is about resources that you received, either directly or indirectly (via your institution), to enable you to complete the work. Checking "No" means that you did the work without receiving any financial support from any third party — that is, the work was supported by funds from the same institution that pays your salary and that institution did not receive third-party funds with which to pay you. If you or your institution received funds from a third party to support the work, such as a government granting agency, charitable foundation, or commercial sponsor, check "Yes." Then complete the provide the information requested.

Section 3. Relevant financial activities outside the submitted work

This section asks about your financial relationships with entities in the biomedical arena that could be perceived to influence, or that give the appearance of potentially influencing, what you wrote in the submitted work. You should disclose interactions with ANY entity that could be considered broadly relevant to the work.

Report all sources of revenue paid (or promised to be paid) directly to you or your institution on your behalf over the 36 months prior to submission of the work. This should include all monies from sources with relevance to the submitted work, not just monies from the entity that sponsored the research. Please note that your interactions with the work's sponsor that are outside the submitted work should also be listed here. If there is any question, it is usually better to disclose a relationship than not to do so.

For grants you have received for work outside the submitted work, you should disclose support ONLY from entities that could be perceived to be affected financially by the published work, such as entities or foundations supported by entities that could be perceived to have a financial stake in the outcome. Public funding sources, such as government agencies, charitable foundations, or academic institutions, need not be disclosed. For example, if a government agency sponsored a study in which you have been involved and drugs were provided by a pharmaceutical company, you need only list the pharmaceutical company.

Section 4. Other relationships

Use this section to report other relationships or activities that readers could perceive to have influenced, or that give the appearance of potentially influencing, what you wrote in the submitted work.

ICMJE Form for Disclosure of Potential Conflicts of Interest

Section 1. Identifying Information

1. Given Name: Abby 2. Surname: Hankins
3. Are you the corresponding author? Yes ___ No_X_
4. Effective Date ________________3/20/22___________
5. Manuscript Title: Biologic Graft Utilization Trends for Rotator Cuff Pathology: Superior Capsular Reconstruction on the Decline

Section 2. The Work Under Consideration for Publication

Did you or your institution at any time receive payment or services from a third party for any aspect of the submitted work (including but not limited to grants, data monitoring board, study design, manuscript preparation, statistical analysis, etc…)?

Complete each item by typing an X in answer yes or not and completing the information requested if an answer is Yes. If you have more than one relationship, add lines.

1. Grant

_X_No ___Yes, money paid to you ___Yes, money paid to institution* Name of entity___ Comments†___

2. Consulting fee or honorarium

_X_No ___Yes, money paid to you ___Yes, money paid to institution* Name of entity___ Comments†___

3. Support for travel to meetings for the study or other purposes

___No _X_Yes, money paid to you
Comments† travel grant from Eastern Virginia Medical School student affairs for Orthopedic Summit 2021 to present research findings

4. Fees for participation in review activities such as data monitoring boards, statistical analysis, end-point committees, and the like

_X_No ___Yes, money paid to you ___Yes, money paid to institution* Name of entity___ Comments†___

5. Payment for writing or reviewing the manuscript

_X_No ___Yes, money paid to you ___Yes, money paid to institution* Name of entity___ Comments†___

6. Provision of writing assistance, medicines, equipment, or administrative support

_X_No ___Yes, money paid to you ___Yes, money paid to institution* Name of entity___ Comments†___

7. Other

_X_No ___Yes, money paid to you ___Yes, money paid to institution* Name of entity___ Comments†___

* This means money that your institution received for your efforts on this study.
† Use this section to provide any needed explanation.

Section 3. Relevant financial activities outside the submitted work

1. Board membership

_X_No ___Yes, money paid to you ___Yes, money paid to institution* Name of entity___ Comments___

2. Consultancy

_X_No ___Yes, money paid to you ___Yes, money paid to institution* Name of entity___ Comments___

3. Employment

_X_No ___Yes, money paid to you ___Yes, money paid to institution* Name of entity___ Comments___

4. Expert testimony

_X_No ___Yes, money paid to you ___Yes, money paid to institution* Name of entity___ Comments___

5. Grants/grants pending

_X_No ___Yes, money paid to you ___Yes, money paid to institution* Name of entity___ Comments___

6. Payment for lectures including service on speakers bureaus

_X_No ___Yes, money paid to you ___Yes, money paid to institution* Name of entity___ Comments___

7. Payment for manuscript preparation

_X_No ___Yes, money paid to you ___Yes, money paid to institution* Name of entity___ Comments___

8. Patents (planned, pending or issued)

_X_No ___Yes, money paid to you ___Yes, money paid to institution* Name of entity___ Comments___

9. Royalties

_X_No ___Yes, money paid to you ___Yes, money paid to institution* Name of entity___ Comments___

10. Payment for development of educational presentations

_X_No ___Yes, money paid to you ___Yes, money paid to institution* Name of entity___ Comments___

11. Stock/stock options

_X_No ___Yes, money paid to you ___Yes, money paid to institution* Name of entity___ Comments___

12. Travel/accommodations/ meeting expenses unrelated to activities listed**

_X_No ___Yes, money paid to you ___Yes, money paid to institution* Name of entity___ Comments___

13. Other (err on the side of full disclosure)

_X_No ___Yes, money paid to you ___Yes, money paid to institution* Name of entity___ Comments___

** For example, if you report a consultancy above there is no need to report travel related to that consultancy on this line.

Section 4. Other relationships

Are there other relationships or activities that readers could perceive to have influenced, or that give the appearance of potentially influencing, what you wrote in the submitted work?

_X_No other relationships/conditions/circumstances that present a potential conflict of interest

___Yes, the following relationships/conditions/circumstances are present (explain below):

>>>>>>>>>>><<<<<<<<<<<

The International Committee of Medical Journal Editors

The ICMJE Disclosure of Potential Conflicts of Interest Form was adopted by *Arthroscopy: The Journal of Arthroscopic and Related Surgery* along with 17 other leading orthopaedic journals at the 2011 annual meeting of the American Academy of Orthopaedic Surgeons.

ICMJE Form for Disclosure of Potential Conflicts of Interest

Section 1. Identifying Information

1. Given Name Kevin 2. Surname Bonner
3. Are you the corresponding author? Yes _X__ No___
4. Effective Date 3/20/22
5. Manuscript Title Biologic Graft Utilization Trends for Rotator Cuff Pathology: Superior Capsular Reconstruction on the Decline

Section 2. The Work Under Consideration for Publication

Did you or your institution at any time receive payment or services from a third party for any aspect of the submitted work (including but not limited to grants, data monitoring board, study design, manuscript preparation, statistical analysis, etc…)?
No

Complete each item by typing an X in answer yes or not and completing the information requested if an answer is Yes. If you have more than one relationship, add lines.

1. Grant

X No ___Yes, money paid to you ___Yes, money paid to institution* Name of entity___ Comments†___

2. Consulting fee or honorarium

X No ___Yes, money paid to you ___Yes, money paid to institution* Name of entity___ Comments†___

3. Support for travel to meetings for the study or other purposes

X No ___Yes, money paid to you ___Yes, money paid to institution* Name of entity___ Comments†___

4. Fees for participation in review activities such as data monitoring boards, statistical analysis, end-point committees, and the like

X No ___Yes, money paid to you ___Yes, money paid to institution* Name of entity___ Comments†___

5. Payment for writing or reviewing the manuscript

X No ___Yes, money paid to you ___Yes, money paid to institution* Name of entity___ Comments†___

6. Provision of writing assistance, medicines, equipment, or administrative support

X No ___Yes, money paid to you ___Yes, money paid to institution* Name of entity___ Comments†___

7. Other

X No ___Yes, money paid to you ___Yes, money paid to institution* Name of entity___ Comments†___

* This means money that your institution received for your efforts on this study.
† Use this section to provide any needed explanation.

Section 3. Relevant financial activities outside the submitted work

1. Board membership

___No _X__Yes, money paid to you ___Yes, money paid to institution* Name of entity
I'm on the Board of the Arthroscopy Assoc of North America -not paid
I'm on the Board of LifeNet Health- paid position

2. Consultancy

___No X Yes, money paid to you ___Yes, money paid to institution* Name of entity___ Comments___
I am a consultant for : LifeNet Health, Depuy/Mitek, Embody

3. Employment

X No ___Yes, money paid to you ___Yes, money paid to institution* Name of entity___ Comments___

4. Expert testimony

X No ___Yes, money paid to you ___Yes, money paid to institution* Name of entity___ Comments___

5. Grants/grants pending

X No ___Yes, money paid to you ___Yes, money paid to institution* Name of entity___ Comments___

6. Payment for lectures including service on speakers bureaus

___No X Yes, money paid to you ___Yes, money paid to institution* Name of entity
Depuy/Mitek

7. Payment for manuscript preparation

X No ___Yes, money paid to you ___Yes, money paid to institution* Name of entity___ Comments___

8. Patents (planned, pending or issued)

___No X Yes, money paid to you ___Yes, money paid to institution* Name of entity
Depuy/Mitek, Embody

9. Royalties

___No X Yes, money paid to you ___Yes, money paid to institution* Name of entity
Depuy/ Mitek, Arthrex, EmbodyPotential Future Royalties- but nothing paid

10. Payment for development of educational presentations

X No ___Yes, money paid to you ___Yes, money paid to institution* Name of entity___ Comments___

11. Stock/stock options

___No X Yes, money paid to you ___Yes, money paid to institution* Name of entity
Embody, COVR Medical

12. Travel/accommodations/ meeting expenses unrelated to activities listed**

X No ___Yes, money paid to you ___Yes, money paid to institution* Name of entity___ Comments___

13. Other (err on the side of full disclosure)

No X Yes, money paid to you ___Yes, money paid to institution* Name of entity___ Comments___

Research Foundation support: Depuy/Mitek, Arthrex, Zimmer

** For example, if you report a consultancy above there is no need to report travel related to that consultancy on this line.

Section 4. Other relationships

Are there other relationships or activities that readers could perceive to have influenced, or that give the appearance of potentially influencing, what you wrote in the submitted work?

X No other relationships/conditions/circumstances that present a potential conflict of interest

___Yes, the following relationships/conditions/circumstances are present (explain below):

>>>>>>>>>>><<<<<<<<<<<

The International Committee of Medical Journal Editors

The ICMJE Disclosure of Potential Conflicts of Interest Form was adopted by *Arthroscopy: The Journal of Arthroscopic and Related Surgery* along with 17 other leading orthopaedic journals at the 2011 annual meeting of the American Academy of Orthopaedic Surgeons.

ICMJE Form for Disclosure of Potential Conflicts of Interest

Section 1. Identifying Information

1. Given Name ____Justin______________________ 2. Surname ______Griffin___________________________
3. Are you the corresponding author? Yes ___ No_x__
4. Effective Date ____3/20/2022_______________________
5. Manuscript Title ___ Biologic Graft Utilization Trends for Rotator Cuff Pathology: Superior Capsular Reconstruction on the Decline

Section 2. The Work Under Consideration for Publication

Did you or your institution at any time receive payment or services from a third party for any aspect of the submitted work (including but not limited to grants, data monitoring board, study design, manuscript preparation, statistical analysis, etc…)?

Complete each item by typing an X in answer yes or not and completing the information requested if an answer is Yes. If you have more than one relationship, add lines.

1. Grant

_x__No ___Yes, money paid to you ___Yes, money paid to institution* Name of entity___ Comments†___

2. Consulting fee or honorarium

__x_No ___Yes, money paid to you ___Yes, money paid to institution* Name of entity___ Comments†___

3. Support for travel to meetings for the study or other purposes

__x_No ___Yes, money paid to you ___Yes, money paid to institution* Name of entity___ Comments†___

4. Fees for participation in review activities such as data monitoring boards, statistical analysis, end-point committees, and the like

_x__No ___Yes, money paid to you ___Yes, money paid to institution* Name of entity___ Comments†___

5. Payment for writing or reviewing the manuscript

_x__No ___Yes, money paid to you ___Yes, money paid to institution* Name of entity___ Comments†___

6. Provision of writing assistance, medicines, equipment, or administrative support

_x__No ___Yes, money paid to you ___Yes, money paid to institution* Name of entity___ Comments†___

7. Other

_x__No ___Yes, money paid to you ___Yes, money paid to institution* Name of entity___ Comments†___

* This means money that your institution received for your efforts on this study.
† Use this section to provide any needed explanation.

Section 3. Relevant financial activities outside the submitted work

1. Board membership

_x__No ___Yes, money paid to you ___Yes, money paid to institution* Name of entity___ Comments___

2. Consultancy

___No _x__Yes, money paid to you ___Yes, money paid to institution* Name of entity_Arthrex Inc__ Comments___

3. Employment

_x__No ___Yes, money paid to you ___Yes, money paid to institution* Name of entity___ Comments___

4. Expert testimony

_x__No ___Yes, money paid to you ___Yes, money paid to institution* Name of entity___ Comments___

5. Grants/grants pending

_x__No ___Yes, money paid to you ___Yes, money paid to institution* Name of entity___ Comments___

6. Payment for lectures including service on speakers bureaus

___No _x__Yes, money paid to you ___Yes, money paid to institution* Name of entity_Arthrex Inc.__ Comments___

7. Payment for manuscript preparation

_x__No ___Yes, money paid to you ___Yes, money paid to institution* Name of entity___ Comments___

8. Patents (planned, pending or issued)

___No _x__Yes, money paid to you ___Yes, money paid to institution* Name of entity_BG Innovations__ Comments___

9. Royalties

___No _x__Yes, money paid to you ___Yes, money paid to institution* Name of entity_Arthrex inc__ Comments___

10. Payment for development of educational presentations

_x__No ___Yes, money paid to you ___Yes, money paid to institution* Name of entity___ Comments___

11. Stock/stock options

_x__No ___Yes, money paid to you ___Yes, money paid to institution* Name of entity___ Comments___

12. Travel/accommodations/ meeting expenses unrelated to activities listed**

_x__No ___Yes, money paid to you ___Yes, money paid to institution* Name of entity___ Comments___

13. Other (err on the side of full disclosure)

_x__No ___Yes, money paid to you ___Yes, money paid to institution* Name of entity___ Comments___

** For example, if you report a consultancy above there is no need to report travel related to that consultancy on this line.

Section 4. Other relationships

Are there other relationships or activities that readers could perceive to have influenced, or that give the appearance of potentially influencing, what you wrote in the submitted work?

_x__No other relationships/conditions/circumstances that present a potential conflict of interest

___Yes, the following relationships/conditions/circumstances are present (explain below):

>>>>>>>>>>><<<<<<<<<<<

The International Committee of Medical Journal Editors

The ICMJE Disclosure of Potential Conflicts of Interest Form was adopted by *Arthroscopy: The Journal of Arthroscopic and Related Surgery* along with 17 other leading orthopaedic journals at the 2011 annual meeting of the American Academy of Orthopaedic Surgeons.

ICMJE Form for Disclosure of Potential Conflicts of Interest

Section 1. Identifying Information

1. Given Name _______John______2. Surname __Taliaferro_______________________________
3. Are you the corresponding author? Yes ___ No_X__
4. Effective Date _________3/20/22__________________

5. Manuscript Title Biologic Graft Utilization Trends for Rotator Cuff Pathology: Superior Capsular Reconstruction on the Decline

Section 2. The Work Under Consideration for Publication

Did you or your institution at any time receive payment or services from a third party for any aspect of the submitted work (including but not limited to grants, data monitoring board, study design, manuscript preparation, statistical analysis, etc...)?

Complete each item by typing an X in answer yes or not and completing the information requested if an answer is Yes. If you have more than one relationship, add lines.

1. Grant

_X__No ___Yes, money paid to you ___Yes, money paid to institution* Name of entity___ Comments†___

2. Consulting fee or honorarium

__X_No ___Yes, money paid to you ___Yes, money paid to institution* Name of entity___ Comments†___

3. Support for travel to meetings for the study or other purposes

_X__No ___Yes, money paid to you ___Yes, money paid to institution* Name of entity___ Comments†___

4. Fees for participation in review activities such as data monitoring boards, statistical analysis, end-point committees, and the like

_X__No ___Yes, money paid to you ___Yes, money paid to institution* Name of entity___ Comments†___ 5. Payment for writing or reviewing the manuscript

X
___No ___Yes, money paid to you ___Yes, money paid to institution* Name of entity___ Comments†___

6. Provision of writing assistance, medicines, equipment, or administrative support

X
___No ___Yes, money paid to you ___Yes, money paid to institution* Name of entity___ Comments†___

7. Other

X
___No ___Yes, money paid to you ___Yes, money paid to institution* Name of entity___ Comments†___

* This means money that your institution received for your efforts on this study. † Use this section to provide any needed explanation.

Section 3. Relevant financial activities outside the submitted work

1. Board membership

X
___No ___Yes, money paid to you ___Yes, money paid to institution* Name of entity___ Comments___

2. Consultancy

X
___No ___Yes, money paid to you ___Yes, money paid to institution* Name of entity___ Comments___

3. Employment
_X__No ___Yes, money paid to you ___Yes, money paid to institution* Name of entity___ Comments___
4. Expert testimony

X
___No ___Yes, money paid to you ___Yes, money paid to institution* Name of entity___ Comments___

5. Grants/grants pending

X
___No ___Yes, money paid to you ___Yes, money paid to institution* Name of entity___ Comments___

6. Payment for lectures including service on speakers bureaus

__X_No ___Yes, money paid to you ___Yes, money paid to 7. Payment for manuscript preparation
_X__No ___Yes, money paid to you ___Yes, money paid to 8. Patents (planned, pending or issued)

_X__No ___Yes, money paid to you ___Yes, money paid to 9. Royalties

X
___No ___Yes, money paid to you ___Yes, money paid to

10. Payment for development of educational presentations
_X__No ___Yes, money paid to you ___Yes, money paid to
11. Stock/stock options
_X__No ___Yes, money paid to you ___Yes, money paid to
12. Travel/accommodations/ meeting expenses unrelated to activities listed**

X
___No ___Yes, money paid to you ___Yes, money paid to institution* Name of entity___ Comments___

13. Other (err on the side of full disclosure)

X
___No ___Yes, money paid to you ___Yes, money paid to institution* Name of entity___ Comments___

** For example, if you report a consultancy above there is no need to report travel related to that consultancy on this line.

institution* Name of entity___ Comments___

institution* Name of entity___ Comments___

institution* Name of entity___ Comments___

institution* Name of entity___ Comments___

institution* Name of entity___ Comments___

institution* Name of entity___ Comments___

Section 4. Other relationships

Are there other relationships or activities that readers could perceive to have influenced, or that give the appearance of potentially influencing, what you wrote in the submitted work?

_X__No other relationships/conditions/circumstances that present a potential conflict of interest ___Yes, the following relationships/conditions/circumstances are present (explain below):

>>>>>>>>>>><<<<<<<<<<<

The International Committee of Medical Journal Editors

The ICMJE Disclosure of Potential Conflicts of Interest Form was adopted by *Arthroscopy: The Journal of Arthroscopic and Related Surgery* along with 17 other leading orthopaedic journals at the 2011 annual meeting of the American Academy of Orthopaedic Surgeons.

ICMJE Form for Disclosure of Potential Conflicts of Interest

Section 1. Identifying Information

1. Given Name Brian 2. Surname Werner
3. Are you the corresponding author? Yes ___ No_X_
4. Effective Date 3/17/2022
5. Manuscript Title: Biologic Graft Utilization Trends for Rotator Cuff Pathology: Superior Capsular Reconstruction on the Decline

Section 2. The Work Under Consideration for Publication

Did you or your institution at any time receive payment or services from a third party for any aspect of the submitted work (including but not limited to grants, data monitoring board, study design, manuscript preparation, statistical analysis, etc…)?

Complete each item by typing an X in answer yes or not and completing the information requested if an answer is Yes. If you have more than one relationship, add lines.

1. Grant

_X_No ___Yes, money paid to you ___Yes, money paid to institution* Name of entity___ Comments†___

2. Consulting fee or honorarium

_X_No __Yes, money paid to you _Yes, money paid to institution* Name of entity Arthrex Comments†___

3. Support for travel to meetings for the study or other purposes

_X_No ___Yes, money paid to you ___Yes, money paid to institution* Name of entity___ Comments†___

4. Fees for participation in review activities such as data monitoring boards, statistical analysis, end-point committees, and the like

_X_No ___Yes, money paid to you ___Yes, money paid to institution* Name of entity___ Comments†___

5. Payment for writing or reviewing the manuscript

_X_No ___Yes, money paid to you ___Yes, money paid to institution* Name of entity___ Comments†___

6. Provision of writing assistance, medicines, equipment, or administrative support

_X_No ___Yes, money paid to you ___Yes, money paid to institution* Name of entity___ Comments†___

7. Other

_X_No ___Yes, money paid to you ___Yes, money paid to institution* Name of entity___ Comments†___

* This means money that your institution received for your efforts on this study.
† Use this section to provide any needed explanation.

Section 3. Relevant financial activities outside the submitted work

1. Board membership

_x_No ___Yes, money paid to you ___Yes, money paid to institution* Name of entity___ Comments___

2. Consultancy

___No _x_Yes, money paid to you _x_Yes, money paid to institution* Name of entity Arthrex Comments___

3. Employment

_x_No ___Yes, money paid to you _Yes, money paid to institution* Name of entity___ Comments___

4. Expert testimony

_x__No ___Yes, money paid to you ___Yes, money paid to institution* Name of entity___ Comments___

5. Grants/grants pending

__x_No ___Yes, money paid to you ___Yes, money paid to institution* Name of entity___ Comments___

6. Payment for lectures including service on speakers bureaus

___No _x_Yes, money paid to you ___Yes, money paid to institution* Name of entity_Arthrex_ Comments___

7. Payment for manuscript preparation

_x_No ___Yes, money paid to you ___Yes, money paid to institution* Name of entity___ Comments___

8. Patents (planned, pending or issued)

_x_No ___Yes, money paid to you ___Yes, money paid to institution* Name of entity___ Comments___

9. Royalties

_x_No ___Yes, money paid to you ___Yes, money paid to institution* Name of entity___ Comments___

10. Payment for development of educational presentations

_x_No ___Yes, money paid to you ___Yes, money paid to institution* Name of entity___ Comments___

11. Stock/stock options

_x_No ___Yes, money paid to you ___Yes, money paid to institution* Name of entity___ Comments___

12. Travel/accommodations/ meeting expenses unrelated to activities listed**

_x_No ___Yes, money paid to you ___Yes, money paid to institution* Name of entity___ Comments___

13. Other (err on the side of full disclosure)

_x_No ___Yes, money paid to you ___Yes, money paid to institution* Name of entity___ Comments___

** For example, if you report a consultancy above there is no need to report travel related to that consultancy on this line.

Section 4. Other relationships

Are there other relationships or activities that readers could perceive to have influenced, or that give the appearance of potentially influencing, what you wrote in the submitted work?

_x_No other relationships/conditions/circumstances that present a potential conflict of interest

___Yes, the following relationships/conditions/circumstances are present (explain below):

>>>>>>>>>>><<<<<<<<<<<

The International Committee of Medical Journal Editors

The ICMJE Disclosure of Potential Conflicts of Interest Form was adopted by *Arthroscopy: The Journal of Arthroscopic and Related Surgery* along with 17 other leading orthopaedic journals at the 2011 annual meeting of the American Academy of Orthopaedic Surgeons.
